# Accelerated Aging during Chronic Oxidative Stress: A Role for PARP-1

**DOI:** 10.1155/2013/680414

**Published:** 2013-11-10

**Authors:** Daniëlle M. P. H. J. Boesten, Joyce M. J. de Vos-Houben, Leen Timmermans, Gertjan J. M. den Hartog, Aalt Bast, Geja J. Hageman

**Affiliations:** Department of Toxicology, Maastricht University, P.O. Box 6200 MD, Maastricht, The Netherlands

## Abstract

Oxidative stress plays a major role in the pathophysiology of chronic inflammatory disease and it has also been linked to accelerated telomere shortening. Telomeres are specialized structures at the ends of linear chromosomes that protect these ends from degradation and fusion. Telomeres shorten with each cell division eventually leading to cellular senescence. Research has shown that poly(ADP-ribose) polymerase-1 (PARP-1) and subtelomeric methylation play a role in telomere stability. We hypothesized that PARP-1 plays a role in accelerated aging in chronic inflammatory diseases due to its role as coactivator of NF-**κ**b and AP-1. Therefore we evaluated the effect of chronic PARP-1 inhibition (by fisetin and minocycline) in human fibroblasts (HF) cultured under normal conditions and under conditions of chronic oxidative stress, induced by *tert*-butyl hydroperoxide (*t*-BHP). Results showed that PARP-1 inhibition under normal culturing conditions accelerated the rate of telomere shortening. However, under conditions of chronic oxidative stress, PARP-1 inhibition did not show accelerated telomere shortening. We also observed a strong correlation between telomere length and subtelomeric methylation status of HF cells. We conclude that chronic PARP-1 inhibition appears to be beneficial in conditions of chronic oxidative stress but may be detrimental under relatively normal conditions.

## 1. Introduction

Chronic inflammatory diseases afflict millions of people across the world leading to a substantial social and economic burden. From diabetes alone, 366 million people worldwide were suffering in 2011 [1]. It is estimated that by the year of 2030 this number will be almost doubled due to the rapid increase in the incidence of the disease caused by population growth, aging, urbanization, and increasing prevalence of obesity and physical inactivity [2]. Chronic inflammatory conditions in diabetes may lead to many serious complications, for example, retinal damage, renal failure, and cardiovascular diseases. Chronic inflammation and chronic oxidative stress, which occur in many chronic diseases, can contribute to the progress of these diseases by accelerating the rate of biological aging [3].

Accelerated biological aging has been associated with telomere shortening [4]. Telomeres are nucleoprotein structures at the end of chromosomes consisting of stretches of a repetitive DNA sequence, TTAGGG in humans. They prevent chromosomal ends from being recognized as double strand breaks and protect them from end-to-end fusions and degradation. In somatic human cells, telomeres shorten with every round of replication (i.e., end replication problem) and cells are triggered into replicative senescence once telomeres shorten to a critical length [3, 5, 6]. However, the end replication problem is not the only factor that contributes to the loss of telomeric DNA. Oxidative stress also appears to play a role in telomere shortening because of the high presence of GGG repeats, which are more readily oxidized compared to a lone guanine in the DNA [7, 8]. Recently, it has been shown that telomeric regions are favoured targets of a persistent DNA damage response induced by genotoxic and oxidative stress, both *in vitro* and *in vivo* [9]. Oxidative stress induces single-strand breaks both directly and indirectly. These are less efficiently repaired in telomeric DNA as compared to genomic DNA and, as a result, increase the rate of telomere shortening due to incomplete replication [10]. 

Since oxidative stress plays a major role in chronic inflammatory diseases, telomere attrition may be involved in the pathophysiology of these diseases. Several studies have linked telomere shortening to various chronic metabolic and inflammatory diseases such as atherosclerosis, diabetes type 2, inflammatory bowel disease, and chronic obstructive pulmonary disease, conditions that are all characterized by the presence of systemic oxidative stress [11–18]. However, the exact underlying mechanisms of telomere shortening under conditions of chronic oxidative stress still have to be elucidated.

Recent evidence indicates that epigenetic regulation may be important in telomere stability. Telomeres lack CpG dinucleotides which are susceptible to methylation, but the immediately adjacent subtelomeric regions have a high density of CpG sequences [19]. In cells deficient in DNA methyltransferases (DNMTs) an induction of telomere elongation was observed which was associated with subtelomeric DNA hypomethylation [20]. Also other studies have found a link between epigenetic status of subtelomeres and telomere length [21], which suggests a role for subtelomeric DNA methylation in telomere stability.

**Figure 1:**
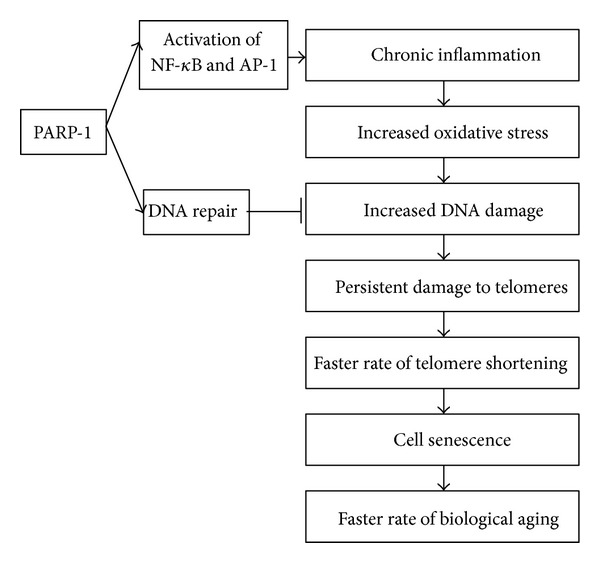
PARP-1 can influence telomere length regulation in two different ways. First, it can enhance DNA repair, protect the telomeres, and contribute to a decrease in the rate of telomere shortening. Second, it can enhance the inflammatory response by activating NF-*κ*B or AP-1, which will lead to more oxidative DNA damage which in turn could accelerate telomere shortening.

The activity of the nuclear enzyme poly(ADP-ribose) polymerase-1 (PARP-1) has also been reported to play a role in this process of telomere stability. It has been shown that PARP-1 associates with telomere repeat binding factor 2 (TRF2), a telomere-specific DNA binding protein that protects chromosome ends by promoting the formation of the “capped” state [22]. Furthermore, PARP-1 has been implicated in the regulation of multiple physiological cellular functions like DNA repair, gene transcription, cell cycle progression, cell death, chromatin function, and genomic stability [23, 24]. PARP-1 may influence telomere stability under conditions of chronic oxidative stress in two different ways (Figure 1). First, through its function in repair of oxidative stress induced DNA damage. It can enhance repair, protect the telomeres, and contribute to a decrease in the rate of telomere shortening. On the other hand, PARP-1 is also a coactivator of the stress-response related transcription factors nuclear factor-kappa B (NF-*κ*B) and activator protein-1 (AP-1), and as a coactivator it can mediate the inflammatory response [25, 26]. In this role it may have a negative effect on telomere shortening, since inflammation will lead to more oxidative stress and oxidative DNA damage which could accelerate telomere shortening. Therefore, it is hypothesized that PARP-1 activity will contribute to accelerated telomere shortening and accelerated aging in chronic inflammatory diseases, through its function as a coactivator of inflammatory responses. The aim of this study was to investigate the effect of chronic PARP-1 inhibition on telomere stability under normal culturing conditions and under conditions of chronic oxidative stress in an *in vitro* model using human fibroblasts (HF). Additionally, the effect of prolonged culturing of HF cells under these conditions on subtelomeric methylation status was studied. 

## 2. Material and Methods

### 2.1. Chemicals

Minimum essential medium (MEM), Hank's buffered salt solution (HBSS), fetal bovine serum (FCS), trypsin, essential amino acids, nonessential amino acids, vitamins, and penicillin/streptomycin were all obtained from Invitrogen (Breda, The Netherlands). Bovine serum albumin (BSA), minocycline, 4′,6-diamidino-2-phenylindole (DAPI), *tert*-butyl hydroperoxide (*t*-BHP), and dimethyl sulfoxide (DMSO) were purchased from Sigma-Aldrich (Zwijndrecht, The Netherlands). The cell supernatant containing mouse monoclonal 10H anti-PAR polymer antibody was produced by Professor W. Buurman (Maastricht University, Maastricht, The Netherlands). FITC-conjugated goat anti-mouse immunoglobulin and fluorescent mounting medium were obtained from DAKO (Glostrup, Denmark). Hydrogen peroxide (H_2_O_2_) was purchased from Merck (Darmstadt, Germany). Fisetin was obtained from Fit Ingredients (Haibach, Germany). Primary human fibroblast cells (normal nonfetal skin tissue) were acquired from Coriell (Coriell Institute for Medical Research, Camden, USA). Hela cell lines were kindly provided by Professor Alexander Bürkle (University of Konstanz, Germany). 

### 2.2. Cell Culture

HF cells were cultured in minimum essential medium (MEM) + GlutaMAX supplemented with 20% nonheat inactivated FCS, 1% penicillin/streptomycin, 0.5% nonessential amino acids, 0.5% essential amino acids, and 0.03% vitamins. Cells were maintained at 37°C in a 5% CO_2_ atmosphere. All cells were passaged at approximately 80% confluency. To induce oxidative stress, parallel cultures were grown with or without exposure to *t*-BHP. To determine the concentration to be used, a concentration series was made from 5 nM to 100 nM *t*-BHP. Cell viability was tested by the trypan blue exclusion test. Cells were viable at concentrations <10 nM. At 5 nM a viability of >80% was found and we used this concentration for our experiments. Additionally, in a separate experiment the effect of PARP-1 inhibition was investigated by supplementation of fisetin (10 mM dissolved in DMSO, further diluted in culture medium to final concentration of 1 *μ*M) or minocycline (10 mM dissolved in DMSO, further diluted in culture medium to final concentration of 100 nM) to the medium of HF cells in the presence or absence of *t*-BHP. All compounds were added to the culture medium every 2-3 days during passaging or medium renewal. The passage at which the experiment was started is called P0.

### 2.3. Immunohistochemical Staining of PAR Polymers

To verify the PARP-1 inhibiting effects of the selected PARP inhibitors, HF cells were seeded at a density of 15 × 10^4^ cells per well in a six-well plate. The next day, cells were treated with 300 *μ*M H_2_O_2_ to induce PARP-1 overactivation and PARpolymer formation. Treatment was done for 10 minutes in the presence or absence of fisetin (1 *μ*M) or minocycline (100 nM), which were added 30 minutes before the H_2_O_2_ treatment. After incubation, the cells were trypsinized, washed with PBS, and fixed in methanol. Fixed cells were put on microscope slides, washed with 0.1% BSA in PBS, and incubated with 100 *μ*L mouse monoclonal 10H anti-PAR polymer antibody for one hour at room temperature. After washing with 0.1% BSA in PBS, cells were incubated with 100 *μ*L polyclonal goat anti-mouse immunoglobulin/FITC for one hour at room temperature. Next, cells were washed again with 0.1% BSA in PBS and incubated for 10 minutes with 100 *μ*L DAPI solution. Subsequently, cells were mounted with fluorescent mounting medium and evaluated using a fluorescence microscope with Lucia GF 4.80 software. At least 100 cells per slide were studied for the presence of PAR polymers in the nucleus.

### 2.4. DNA Isolation

DNA was extracted using the QIAamp DNA Mini Kit (Qiagen, Venlo, The Netherlands) according to the manufacturer's protocol and quantified using a Nano-drop (Isogen Life Science, Belgium).

### 2.5. Telomere Length Measurement

Telomere length was determined by quantitative PCR as previously described [11, 27]. Two master mixes were prepared, one with telomere primers and one with human *β* globin (HBG) primers (1x iQ SYBR Green Supermix from Bio-Rad). Sequences and concentrations of the primers are shown in Table 1. Sample DNA was pipetted in a 96-well plate at a final concentration of 10 ng/*μ*L. 20 *μ*L of the mastermix was added and the plate was shortly centrifuged. Each sample was run in triplicate. For the standard curve a reference DNA sample was diluted serially to produce three concentrations of 1.25, 5, and 10 ng/*μ*L. In every run, negative controls (MQ + mastermix) and reference samples were included. The references were derived from two different Hela cell lines, one with relatively short telomeres (Hela S3: 5.5 kb) and one with long telomeres (Hela 229: 14-15 kb). By adding reference DNA of controls to each qPCR, a standard curve could be created and the absolute telomere length of the samples could be calculated as kilo-base pairs (kbp). Hela cell lines were kindly provided by Professor Alexander Bürkle, University of Konstanz, Germany. The PCR was performed using a Bio-Rad MyiQ iCycler Single Color RT-PCR detection system using iQ SYBR Green Supermix, containing iTaq polymerase, dNTPs, SYBR Green I, and buffers (Bio-Rad, CA, USA).

### 2.6. Telomerase Activity

Telomerase activity was evaluated by telomeric repeat amplification protocol (TRAP) assay using the TeloTAGGG Telomerase PCR ELISAPLUS (Roche Diagnostics, Milan, Italy) which is an extension of the original method described by Kim et al. [28]. Cell extracts (1–3 × 10^5^ cell equivalents) were employed in the first step, in which telomerase adds telomeric repeats (TTAGGG) to the 3′-end of the biotin-labeled synthetic P1-TS primer. These elongation products, as well as the internal standard (IS), were amplified by PCR. In the second step the PCR products were split into two aliquots, denatured, denatured and hybridized separately to digoxigenin-(DIG)-labeled detection probes, specific for the telomeric repeats and for the IS, respectively. Results obtained by densitometric analysis were normalized upon the data of IS and expressed as relative telomerase activities (RTA).

### 2.7. Subtelomere Methylation

Bisulfite treatment of genomic DNA was performed with an EZ DNA methylation kit (Zymo Research, CA, USA). The bisulfite-treated DNA was subjected to a polymerase chain reaction (PCR) to amplify the subtelomere region with the primers specific for chromosome arm 2p. Primer sequences were obtained from Lee et al. [29]. Methylation specific primers were: forward ATGATTAGCGAGTTCGGTTTTAAC and reverse: GAATCGCGCCAAATATATACG, and primers specific for unmethylated DNA were forward: GATGATTAGTGAGTTTGGTTTTAATG and reverse: ACAAATCACACCAAATATATACAAA. PCR reactions were performed in a total volume of 25 *μ*L containing 1x Taq polymerase buffer, 2 mM MgCl_2_, 0.2 mM dNTPs, 0.6 *μ*M of each primer, 1 U Taq polymerase, and 500 ng bisulfite-treated DNA. PCR amplification was conducted as follows: initial denaturation of 95°C for 10 minutes, followed by 40 cycles of 94°C for 30 seconds, 58°C for 30 seconds and 72°C for 30 seconds, and ending with an extension at 72°C for 10 minutes. Amplified products were run on an ethidium bromide stained with 2% agarose gel. Quantification was done by measuring grey values with the program ImageJ (http://rsbweb.nih.gov/ij/).

### 2.8. Statistical Analysis

Differences between groups for telomere length and PAR polymer staining were tested using the Mann-Whitney *U* test. Effects of PARP-1 inhibition were tested using a Wilcoxon signed-rank test. The association between telomere length and subtelomeric methylation status was evaluated using the nonparametric Spearman's rank correlation coefficient. *P* values < 0.05 were considered statistically significant and *P* values < 0.1 were considered statistical trends. Statistical analyses were analyzed with SPSS for Windows (version 20.0; SPSS Inc., Chicago, IL, USA).

## 3. Results

**Figure 2:**
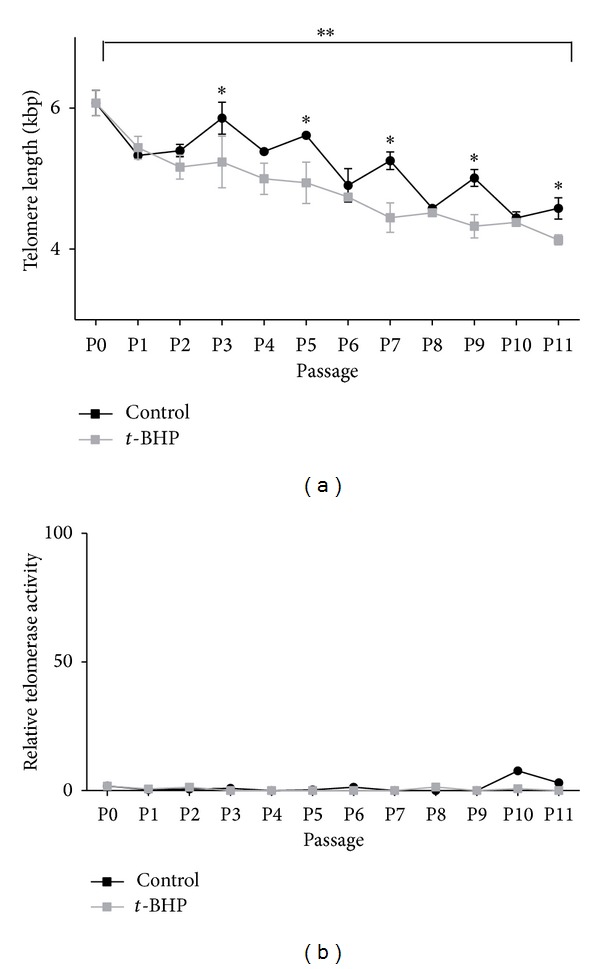
(a) Telomere length and (b) telomerase activity in human fibroblasts during culturing in the absence (black line) or presence (grey line) of 5 nM *tert*-butyl hydroperoxide (*t*-BHP). HF cells exposed to *t*-BHP showed significantly shorter telomeres than nonexposed cells of the same passage number. Telomere length significantly decreased over time in nonexposed cells as well as in exposed cells. **P < 0.001 compared to P0 (start of the experiment); *P < 0.01 compared to nonexposed cells of the same passage number. Mean ± SD of telomere length is shown. If telomerase activity was present, the relative telomerase activity value would be higher than 100. The values are lower than 10, which indicates that there is no telomerase activity in these cells.

To determine whether chronic exposure of HF cells to oxidative stress induces a faster rate of telomere shortening, we determined telomere length of HF cells exposed to 5 nM *t*-BHP. HF cells exposed to *t*-BHP showed significantly shorter telomeres than nonexposed cells of the same passage number (*P* < 0.01) (Figure 2(a)). It was also observed that the telomere length significantly decreased over time in nonexposed cells as well as in exposed cells (*P* < 0.001) (Figure 2(a)). In addition, the population doubling time was increased in the exposed cells compared to nonexposed cells. Telomere length decreased with approximately 1490 bp after 45 population doublings in the nonexposed cells and with 1938 bp after 45 population doublings in the exposed cells. In the nonexposed cells, at several time points the telomere length appeared to increase instead of decreasing. To be able to explain this phenomenon we measured the telomerase activity in the cells. As expected (as we used a primary cell line), no detectable telomerase activity was measured (Figure 2(b)).

**Figure 3:**
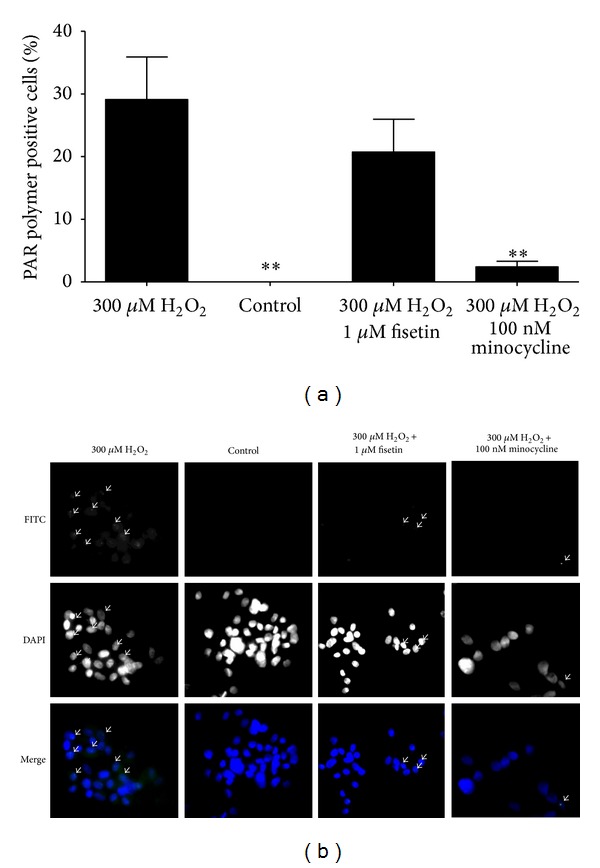
(a) PAR polymer formation in human fibroblasts treated with 300 *μ*M H_2_O_2_ for 10 minutes in the presence or absence of 1 *μ*M fisetin or 100 nM minocycline which were added 30 minutes before the H_2_O_2_ treatment. **P < 0.05 compared to cells treated with 300 *μ*M H_2_O_2_. (b) Representative photographs of PAR polymer staining. Arrows indicate examples of PAR polymer positive cells. Magnification: 300x.

To confirm the PARP-1 inhibiting effect of fisetin and minocycline, HF cells were treated with H_2_O_2_ to induce PARP activity. The formation of PAR polymers in these cells was evaluated using immunohistochemical staining. In nontreated cells, no PAR polymer formation was observed. Treatment with H_2_O_2_ induced an increase in the number of PAR polymer positive cells (*P* < 0.01). Preincubation with fisetin decreased the number of PAR polymer positive cells with 40% indicating that 1 *μ*M fisetin mildly inhibits PARP-1. Preincubation with 100 nM minocycline resulted in a 90% reduction in the number of PAR polymer positive cells (*P* < 0.001), indicating that minocycline is a strong inhibitor of PARP-1 (Figure 3).

**Figure 4:**
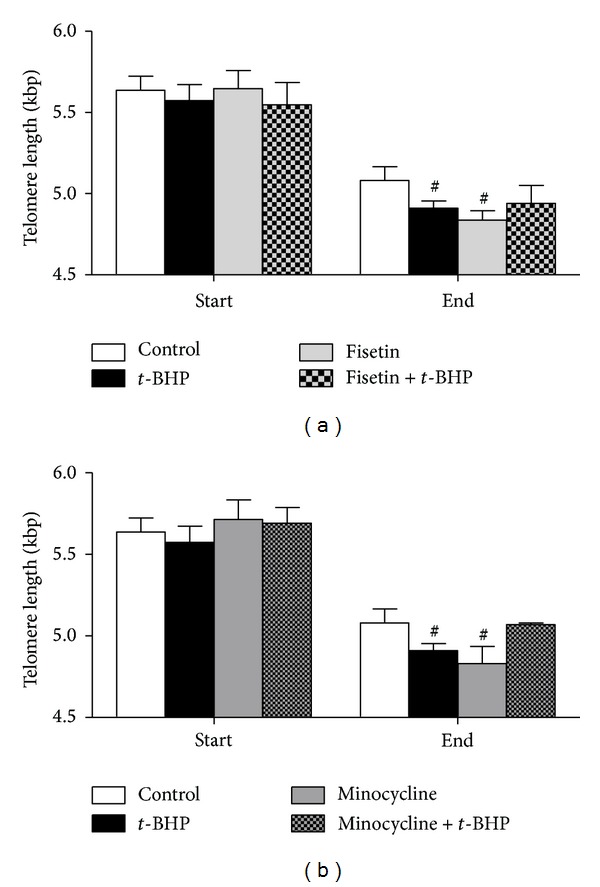
Effect of PARP inhibition by 1 *μ*M fisetin (a) and 100 nM minocycline (b) on telomere length at the start (P0–P2) and end (P9–P11) of the experiment cultured under normal conditions or under conditions of chronic oxidative stress induced by* tert*-butyl hydroperoxide (*t*-BHP). ^#^ *P* < 0.1 compared to control.

To investigate the effect of chronic PARP-1 inhibition on telomere length regulation under conditions of chronic oxidative stress, HF cells were cultured with 1 *μ*M fisetin or 100 nM minocycline in the presence or absence of *t*-BHP. After 10 passages, telomeres in all culturing conditions were shorter compared to telomere length at the start of the experiment (Figure 4). In addition, culturing the cells in presence of *t*-BHP, fisetin, and minocycline (*P* < 0.1) resulted in shorter telomeres compared to untreated cells. However, culturing them in presence of minocycline or fisetin in combination with *t*-BHP did not result in accelerated telomere shortening when compared to untreated cells (Figure 4). Additionally, at the end of the experiment, cells treated with *t*-BHP, minocycline and *t*-BHP, and fisetin showed a senescence-like phenotype (flattened, contracted, and detached cells [30]). Cells cultured under the other conditions had a normal appearance. Also, cells cultured with *t*-BHP, *t*-BHP and fisetin, and minocycline alone showed a decreased growth rate, resulting in a longer period before passaging (Table 2).

**Figure 5:**
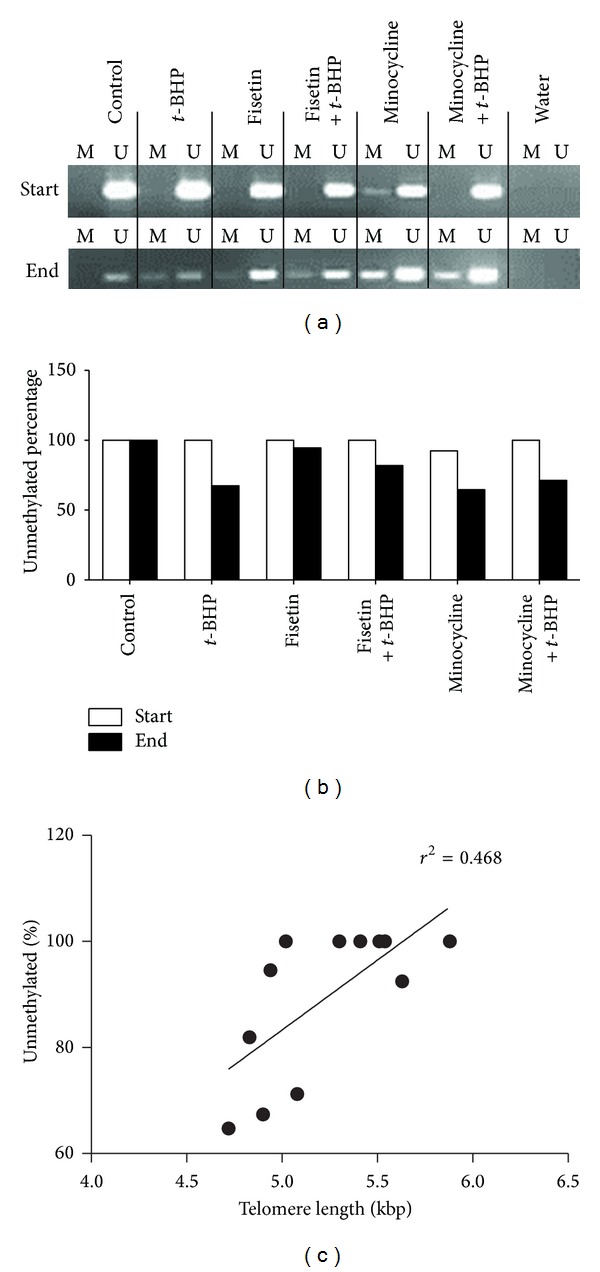
(a) Methylation specific PCR (MSP) result for chromosome 2p at passage 1 (start) and passage 10 (end). M and U indicate amplification from methylation and unmethylation sequence-specific primers. Water indicates MSP result with no template DNA (negative control). Densitometry of the photographs is shown in B. (c) Spearman's correlation revealed a statistically significant correlation (*r*_*s*_ = 0.668; *P* = 0.018) between telomere length and the level of methylation.

To examine the effect of subtelomeric methylation on telomere stability, the methylation status of chromosome 2p was evaluated at the start and end of the experiment (Figure 5). At the start of the experiment HF cells showed an unmethylated pattern of the subtelomere region of chromosome 2p. At the end of the experiment differences between the conditions were observed. Cells cultured under normal conditions showed a methylation pattern that was similar to the pattern at the start of the experiment, while cells treated with *t*-BHP or minocycline showed an increase in methylation of almost 30%. For cells treated with fisetin this increase was only half (~15%). Spearman's correlation was run to determine the relationship between the level of methylation and telomere length, which revealed a statistically significant correlation (*r*_*s*_ = 0.668; *P* = 0.018). 

## 4. Discussion

In this study we investigated the effect of chronic PARP-1 inhibition on telomere stability under conditions of chronic oxidative stress. We used prolonged culturing of HF as a model for development of cellular senescence. To induce oxidative stress, we cultured HF in presence of *t*-BHP, which is a short-chain organic hydroperoxide that produces free radicals after metabolic activation [31].

### 4.1. Chronic Oxidative Stress Induces Telomere Shortening

Prolonged culturing of HF resulted in telomeres shortening, indicating biological aging of HF. In addition, exposure to chronic oxidative stress significantly increased the rate of telomere shortening. Because the observed telomere shortening might be caused by an increased rate of cell division, the population doubling times were calculated. We found that the population doubling time was increased in the cells exposed to *t*-BHP, which could be caused by an increased level of apoptosis, leading to a decreased cell proliferation capacity. Unexpectedly, at several time points in the nonexposed cells telomere length was increased. This appeared not to be caused by increased telomerase activity, since telomerase activity was absent or very low in this primary cell line. An alternative mechanism may be involved which can be adopted by yeast and human telomerase-deficient cell lines, the alternative lengthening of telomeres (ALT) for telomere maintenance. ALT appears to be mechanistically related to survival in cells and to involve a homologous recombination based mechanism in which one telomere can be extended using the telomere from a nonhomologous chromosome arm or extrachromosomal telomeric DNA [32–34]. This mechanism has been demonstrated to exist in HF [35, 36]. Other processes may contribute to the observed increase in telomere length as well, such as survival and selection of cells with longer telomeres and with a better adaptation to the culturing conditions. 

### 4.2. Inhibition of PARP-1 by Fisetin and Minocycline

Fisetin and minocycline were used to inhibit PARP-1. Fisetin is a flavonoid that is normally present in dietary sources like fruits and vegetables [37]. Fisetin has been described to have many beneficial health effects, like memory enhancement [38]. It was found to possess anti-inflammatory effects via inhibition of the activation of NF-*κ*B [39] and it has been shown previously that fisetin inhibited PARP-1 in pulmonary epithelial cells [40]. 

At a concentration of 1 *μ*M, fisetin caused a mild inhibition of PARP-1 activity when cells were exposed to H_2_O_2_. Chronic treatment of HF cells with fisetin resulted in shorter telomeres compared to control cells. In cells cultured in the presence of both fisetin and *t*-BHP average telomere length was not significantly different compared to that of control cells. This finding appeared to support our hypothesis of the involvement of PARP-1 activity in telomere stability. 

A recent study by Sengupta et al. showed fisetin to be a promising ligand for the formation of a four-stranded structure known as a G-quadruplex [41]. Formation of quadruplexes has been shown to decrease the activity of telomerase, but it also inhibits the ALT mechanism [42, 43]. Since this mechanism might play a role in our model, it is possible that quadruplex formation by fisetin under normal situations causes a faster rate of telomere shortening due to impairment of ALT. G-quadruplex formation is beneficial in anti cancer therapy, as it impedes telomere elongation, a mechanism most tumor cells use for unlimited proliferation [44]. 

Minocycline, also known as minocycline hydrochloride, is a member of the broad spectrum tetracyclines antibiotics. It is primarily used to treat acne and other skin infections and exerts anti-inflammatory effects that are completely separate from its antimicrobial actions [45]. We showed that nanomolar concentrations of minocycline significantly inhibited PARP-1 activity, as was previously reported by Alano et al. [46]. Culturing of cells in the presence of 100 nM minocycline resulted in shorter average telomere length as compared to control cells. On the other hand, when cells were cultured in the presence of minocycline in combination with *t*-BHP, average telomere length was not significantly decreased when compared to control. These findings, together with the findings of inhibition by fisetin, appeared to support our hypothesis that PARP-1 activity contributes to telomere stability and that inhibition of PARP-1 activity increases the rate of telomere shortening. However, under conditions of chronic oxidative stress, inhibition of PARP-1 appeared to result in a decreased rate of telomere shortening. The anti-inflammatory activity of fisetin and minocycline may also contribute to the stabilizing effect on telomere length under chronic oxidative stress conditions.

The effects of chronic minocycline treatment on mammalian cells are still largely unknown. Research mainly focussed on examining the possible neuroprotective and anti-inflammatory effects of minocycline on progression of neurodegenerative disorders like multiple sclerosis (MS) and amyotrophic lateral sclerosis (ALS). It has been described that minocycline is effective in various experimental models of ALS, Parkinson and Huntington disease [47–51]. In a study of patients with acute stroke it was found that treatment with minocycline significantly improved the outcome compared to patients treated with placebo [52]. Since oxidative stress is known to play a role in neuronal cell death in these diseases [53], PARP-1 inhibition might be an underlying mechanism by which minocycline exerts these neuroprotective effects. However, minocycline has also been shown to be effective in other disease models. It was already found that minocycline may prevent blindness in a rat model of diabetic retinopathy [45]. Several studies have suggested an important role of PARP activation in the pathogenesis of diabetic complications like nephropathy, neuropathy, and retinopathy [54–56]. Furthermore, minocycline has been shown to exert *in vivo* cardioprotective effects by suppressing oxidative stress and therefore preventing fetal cardiac myocyte death after prenatal cocaine exposure [57]. 

A selective PARP-1 inhibitor was not tested in our experiments. However, Beneke et al. described an experiment in which they exposed cells to the well-known PARP-1 inhibitor 3-aminobenzamide (3-AB) and measured telomere length [58]. They found in two mammalian cell systems (hamster and human) that pharmacological inhibition of PARP1 led to a fast, dose-dependent decrease of telomere length. These results are comparable with the results we obtained in our experiment in which fisetin and minocycline were applied as PARP inhibitors.

The effects on telomere length were observed to be similar with both inhibitors. However, minocycline treated cells showed morphological changes at an earlier passage than untreated cells or cells treated with fisetin. This might be explained by the fact that minocycline is known to have strong PARP-1 inhibiting capacity only, whereas fisetin has been reported to exert other effects that may enhance cellular function [37]. Fisetin is an activator of sirtuin 1 (SIRT1), a histone deacetylase [59]. An increased activity of SIRT1 is associated with enhanced survival and longevity. Fisetin has already been shown to increase the lifespan of the yeast *Saccharomyces cerevisiae* [59]. Additionally, fisetin is able to inhibit COX2 expression [60]. It has been shown that selective COX2 inhibitors can modulate cellular senescence in human dermal fibroblasts [61]. Activation of SIRT1 and/or inhibition of COX2 by fisetin could explain why cells treated with fisetin had a normal appearance and cells treated with minocycline, which is not known as a SIRT1 activator, were in a senescence state at passage 11, although they had similar telomere length. 

### 4.3. Subtelomeric Methylation

Mouse models and *in vitro* studies suggest a role for subtelomeric methylation in telomere length regulation [20, 21]. Normally, telomeres have a “closed” conformation which is established by epigenetic markers, including methylation of the subtelomeric region. When telomeres become shorter, the epigenetic markers decrease, which leads to a more “open” confirmation that allows a greater accessibility for telomere-elongating activities [62]. It has been shown that the subtelomeric region of Alzheimer patients with short telomeres was hypermethylated [63]. In contrast, in patients with Parkinson's disease an increase in short telomeres with subtelomeric hypomethylation was found [64]. PARP-1 is also known to be able to influence DNA methylation by regulation the expression and activity of DNMT1 or by direct interaction with DNMT1 [65–67]. We observed a correlation between methylation status and telomere length. We found a change from unmethylated status at the beginning of the experiment to 50% methylated in conditions with the shortest telomeres at the end of the experiment of the subtelomeric region of chromosome 2p. Cells that were chronically treated with fisetin showed less increase in methylation, which could be caused by the ability of fisetin to inhibit SssI DNMT- and DNMT1-mediated DNA methylation [68]. 

## 5. Conclusion

Chronic fisetin treatment of HF at physiological concentrations resulted in shorter telomeres compared to control cells, indicating reduced telomere stability and enhanced biological aging of these cells. Under the assumption that it is healthy, fisetin is often added to nutritional supplements at relatively high concentrations. Since the biological effects of regular consumption of high doses of fisetin (and also flavonoids in general) are not known, thorough safety evaluation is warranted with respect to these nutritional supplements. Chronic minocycline treatment also enhanced telomere shortening. This implies that precaution should be taken when minocycline is subscribed as a chronic treatment. 

However, under conditions of chronic oxidative stress, both fisetin and minocycline appeared to reduce the rate of telomere shortening. Since our study was limited to testing the effects of fisetin and minocycline in an *in vitro* model with HF cells that were chronically exposed to oxidative stress more research is needed to evaluate possible positive effects of fisetin and minocycline in chronic inflammatory diseases. 

It can be concluded that chronic administration of pharmaceuticals or nutraceuticals with PARP inhibiting activity appears to be beneficial in conditions of chronic oxidative stress, but may be detrimental under relatively normal conditions.

## Figures and Tables

**Table 1:** Primer sequences and concentrations for telomere length PCR.

Primer	Sequence (5′ to 3′)	Concentration
Telomere 1	CGGTTTGTTTGGGTTTGGGTTTGGGTTTGGGTTTGGGTT	100
Telomere 2	GGCTTGCCTTACCCTTACCCTTACCCTTACCCTTACCCT	900
HBG forward	GCTTCTGACACAACTGTGTTCACTAGC	300
HBG reverse	CACCAACTTCATCCACGTTCACC	700

**Table 2:** Average time span to reach 80% confluency in HF cultures (moment of passage). Mean ± SD are shown.

Condition	Days between passage (average P0–P11)
Control	5.5 ± 1.0
*t*-BHP	6.1 ± 1.2
Fisetin	5.6 ± 1.0
Fisetin + *t*-BHP	6.7 ± 2.4
Minocycline	6.2 ± 2.2
Minocycline + *t*-BHP	6.1 ± 1.0
